# The Loop2 Insertion of Type IX Myosin Acts as an Electrostatic Actin Tether that Permits Processive Movement

**DOI:** 10.1371/journal.pone.0084874

**Published:** 2014-01-09

**Authors:** Kerstin Elfrink, Wanqin Liao, Uwe Pieper, Stefanie J. Oeding, Martin Bähler

**Affiliations:** Institute of Molecular Cell Biology, Westfalian Wilhelms-University, Muenster, Germany; Stanford University School of Medicine, United States of America

## Abstract

Although class IX myosins are single-headed, they demonstrate characteristics of processive movement along actin filaments. Double-headed myosins that move processively along actin filaments achieve this by successive binding of the two heads in a hand-over-hand mechanism. This mechanism, obviously, cannot operate in single-headed myosins. However, it has been proposed that a long class IX specific insertion in the myosin head domain at loop2 acts as an F-actin tether, allowing for single-headed processive movement. Here, we tested this proposal directly by analysing the movement of deletion constructs of the class IX myosin from *Caenorhabditis elegans* (Myo IX). Deletion of the large basic loop2 insertion led to a loss of processive behaviour, while deletion of the N-terminal head extension, a second unique domain of class IX myosins, did not influence the motility of Myo IX. The processive behaviour of Myo IX is also abolished with increasing salt concentrations. These observations directly demonstrate that the insertion located in loop2 acts as an electrostatic actin tether during movement of Myo IX along the actin track.

## Introduction

Myosins are actin dependent motor molecules that can convert chemical energy from ATP-hydrolysis into mechanical force along actin filaments. Myosins are involved in many different dynamic cellular processes such as cell differentiation, cell migration or maintenance of cell morphology [1]–[3]. All myosins share a common domain structure consisting of three regions: a head region, a neck region and a tail region. The N-terminal head region has a conserved sequence and contains the nucleotide- and actin-binding sites. The neck region is capable of binding variable numbers of light chains. It acts as a lever arm amplifying small structural changes that are produced within the head. Finally, the tail region is the most variable domain. It is responsible for harnessing motor functions for different cellular processes. Based on sequence homologies of the head region myosins have been assigned to 47 different classes which makes myosins one of the largest and most divergent protein families known (http://www.cymobase.org).

Most vertebrates contain two genes for class IX myosins while in invertebrates a single gene for class IX myosins has been identified. The two class IX myosins in mammals, myosin IXa (Myo9a, myr 7) and myosin IXb (Myo9b, myr 5), exist in multiple splice variants [4]. In the tail region class IX myosins carry one or two C1 zinc binding domains and a Rho GTPase-activating protein (RhoGAP) domain that inactivates the small GTPase Rho. Hence, myosin IX can be considered as a motorized signaling molecule [4], [5]. Mice deficient in myosin IXa develop severe hydrocephalus and it has been shown that myosin IXa plays an important role in epithelial differentiation and morphology [6]. Myosin IXb deficient mice on the other hand do not exhibit a clear phenotype. However, it has been demonstrated that myosin IXb regulates the migration of macrophages and possibly other immune cells [7].

Based on their motor properties myosins can be classified into two groups: the ones that move processively along actin filaments and the ones that do not move processively. Processivity is defined as the ability to perform multiple successive steps along a track without dissociation. Such processive movement can be achieved when a particular myosin spends a large fraction of the total ATPase cycle time firmly attached to F-actin. Such a myosin is called a high duty ratio motor. Dimerisation of a high duty ratio motor then allows for processive hand-over-hand movement. Coordination of the chemical cycles in the two motor domains by mechanical strain further improves processivity. Surprisingly, it has been observed that mammalian myosin IXb and myosin IX from *Caenorhabditis elegans* (*C. elegans*) belong to the group of processive motor molecules although they are single-headed [10]–[12]. This implies that class IX myosins have developed a mechanism for single-headed processive movement that differs from the hand-over-hand mechanism described for double-headed myosins [13], [14]. Concomitantly class IX myosins exhibit some unique features with respect to their catalytic cycle. Unlike in any other characterized myosin, ATP hydrolysis is the rate-limiting step in the ATPase cycle. This is remarkable, because in other myosin classes the ATP-bound state has a low affinity to F-actin [15], [16]. However, it turns out that myosin IXb has a considerably high affinity for actin in the ATP-bound state compatible with processivity [17]-[19]. Therefore, it was proposed that myosin IX carries an additional actin-binding site within the head region that can serve as an actin tether during processive movement [17], [20], [21]. Class IX myosins carry two unique domains in the head region that are not present in other myosins. Namely, the head contains an extension located at the N-terminus and a large basic insertion located in the loop2 region. The unique motor properties might depend on these two extra domains. Indeed, deletion of the loop2 insertion of rat myosin IXb decreases its actin affinity considerably and the isolated loop2 insertion binds to actin filaments with high affinity [22].

In order to analyse the motor properties of class IX myosins, we set out to study myosin IX from *C. elegans* (Myo IX). This invertebrate class IX myosin is more closely related to mammalian myosin IXb than myosin IXa [23]. Previously we described that the Myo IX head region demonstrates characteristics of a processive motor and moves towards the plus-end of actin filaments [12]. To examine whether the two unique domains in the head region of class IX myosins define the motor properties, we created and analysed three different Myo IX constructs: The wildtype motor domain (Myo IX head), the motor domain lacking the N-terminal extension (Myo IX headΔext), and the motor domain lacking the loop2 insertion (Myo IX headΔins). Here we report that Myo IX headΔins no longer shows characteristics of a processive motor while Myo IX headΔext still does. Furthermore, we show that increasing ionic strength leads to loss of processive behaviour of the Myo IX head. From these data we conclude that the loop2 insertion acts as an electrostatic actin tether during movement of Myo IX.

## Results

### Purification of biotinated myosin IX head constructs

**Figure 1:**
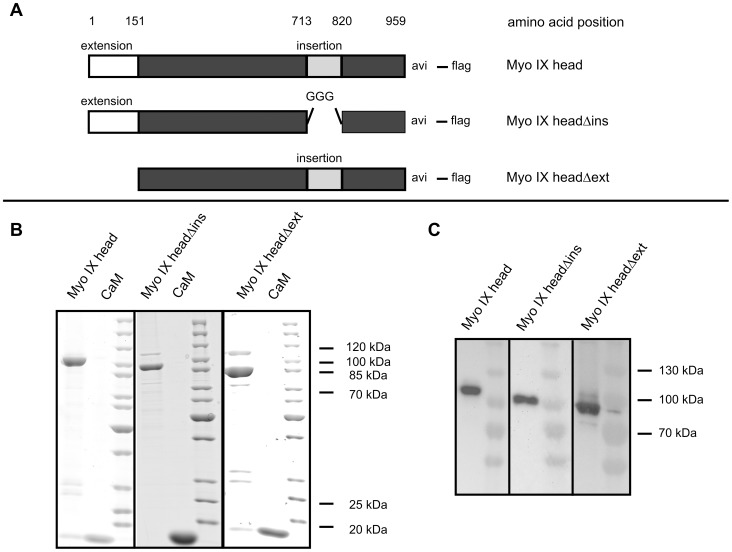
Purification of myosin IX head constructs. Different Myo IX head constructs as shown in panel **A** were purified by α-flag affinity chromatography from Sf9-cells and separated by 10–15% gradient SDS-PAGE. Proteins were either stained by coomassie blue (**B**) or transferred to a PVDF membrane followed by specific detection of biotinated proteins by HRP-coupled streptavidin (**C**). CaM  =  recombinant calmodulin.

In order to study the contributions of the N-terminal extension and of the loop2 insertion to the motor properties of Myo IX, three different Myo IX constructs (shown schematically in Fig. 1A), Myo IX head (113 kDa), Myo IX headΔins (101 kDa) and Myo IX headΔext (96 kDa) were purified by Flag-antibody affinity chromatography (Fig. 1B). Purification yielded average concentrations of 2.1 µM Myo IX head with a purity of 78% and of 1.8 µM Myo IX headΔins and Myo IX headΔext with purities of 77%. As described previously, calmodulin co-purifies with Myo IX head. Based on calmodulin overlay assays the calmodulin binding site was assigned to the N-terminus of the loop2 insertion [12]. In agreement with these findings, calmodulin was co-purified with Myo IX headΔext, but not with Myo IX headΔins (Fig. 1B). The major impurities in the purified Myo IX constructs corresponded to degradation products as determined by LC-MS (data not shown).

For characterization of the motor properties by *in vitro* motility assays Myo IX head constructs were biotinated. In addition to baculoviruses encoding the corresponding Myo IX head construct and calmodulin, Sf9 cells were co-infected with a baculovirus encoding the BirA biotin ligase. To verify the *in vivo* biotination of the constructs at the C-terminal avi tag, Western blot experiments were performed and biotinated proteins were detected with HRP-coupled streptavidin. Fig. 1C shows that Myo IX head, Myo IX headΔins and Myo IX headΔext became biotinated in the infected Sf9 cells.

### Size distribution of purified myosin IX head

**Figure 2:**
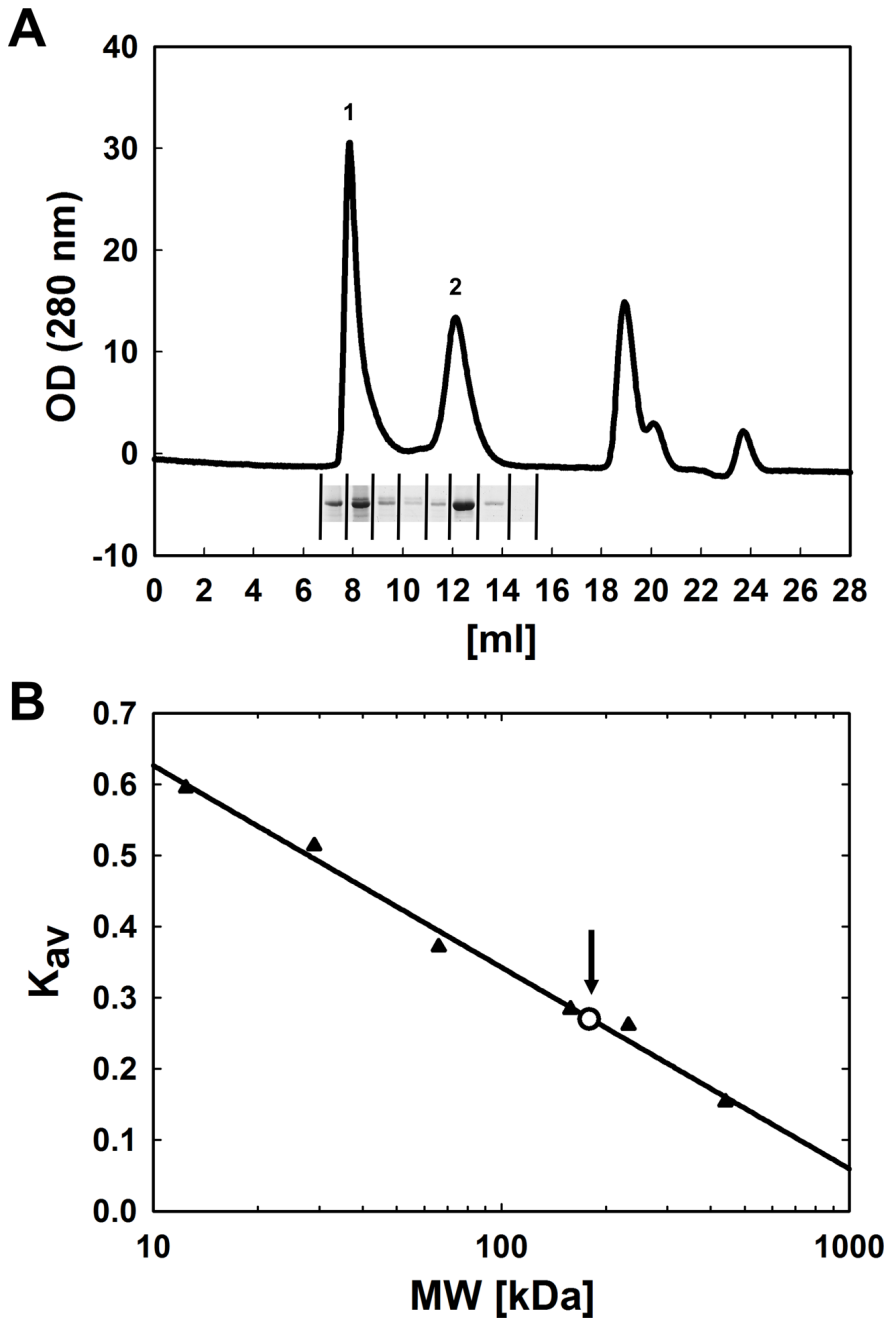
Size distribution of purified myosn IX head. A volume of 200 µl purified Myo IX head was loaded on a size exclusion column (Superdex 200; 10/300 GL) in assay buffer including 130 mM KCl. **A:** Optical density was monitored at 280 nm and plotted against the elution volume. Myo IX head could be detected by SDS-PAGE in peak 1 and peak 2 as shown. **B:** The column was calibrated by the use of standard proteins (closed triangles) and K_av_ was calculated (equation 1). The arrow marks K_av_ determined for peak 2 (open circle) that corresponds to a molecular weight of 192 kDa.

In order to analyse the molecular state of purified Myo IX head, we performed size exclusion chromatography. The elution profile of MyoIX head was characterized by three peaks (Fig. 2). The Myo IX head was present in peak 1 and peak 2 as detected by SDS-PAGE after precipitation. Peak 3 contained the FLAG-peptide. The elution volume of peak 1 corresponded approximately to the void volume of the column. Thus, the molecular weight was ≥1300 kDa (the exclusion limit of the column) showing that Myo IX head in this peak is multimeric. In contrast, the molecular weight of Myo IX head eluted in peak 2 was determined to be 192±4.3 kDa from nine independent experiments. For a 1∶1 complex of monomeric Myo IX head and calmodulin a molecular weight of 130 kDa would be expected. Because dimeric Myo IX head has a molecular weight of a minimum of 226 kDa when no calmodulin is bound and of 260 kDa when one calmodulin is bound per Myo IX head, we can safely assume that the population of Myo IX head eluted in peak 2 reflects monomeric, albeit not perfectly globular, Myo IX head in complex with calmodulin.

### In vitro filament gliding driven by myosin IX head constructs

**Figure 3:**
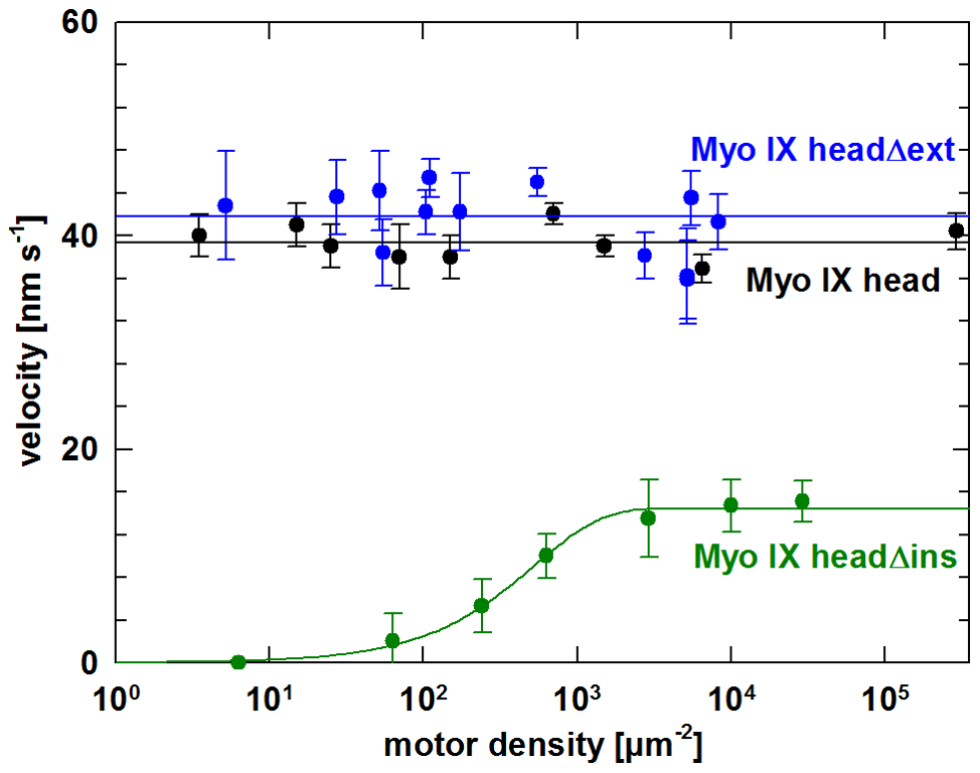
F-actin gliding velocity driven by myosin IX head constructs. Filament gliding assays were performed as described in experimental procedures. Varying amounts of purified Myo IX head (black), Myo IX headΔins (green) or Myo IX headΔext (blue) were immobilized specifically by biotin-streptavidin interaction on the surface of a flow cell. Velocity of F-actin gliding was determined at different motor densities on the surface. The velocity as a function of motor density was fitted using equation 2. Errors presented are standard deviations of varying numbers of tracked filaments. Exemplary movies for each construct can be found under supporting information (movies S3–S5).

To induce movement of actin filaments, Myo IX needed specific attachment to the surface. Biotinated Myo IX constructs were coupled to a surface covered with biotinated BSA and streptavidin. After addition of fluorescently labelled F-actin and ATP, actin filament gliding was monitored. Since two different populations of Myo IX head, i.e. a monomeric and a multimeric population were present in our initial preparations, the question came up which population was responsible for the processive behaviour of Myo IX head that we had observed in a former study [12]. To address this question, we analysed the actin filament gliding activity of both populations at an ionic strength of 30 mM KCl. Thereby we observed that the population consisting of monomeric Myo IX head showed filament gliding activity while the other population consisting of multimeric Myo IX head did not support gliding nor binding of F-actin (movies S1 and S2). Therefore, only monomeric Myo IX head is able to support actin filament gliding and the multimers are not interfering in any way with the gliding assay. Nevertheless, all further experiments were performed with Myo IX constructs that had been pre-cleared by ultracentrifugation immediately before use. Under these conditions actin filament gliding was observed for all three Myo IX constructs (movies S3–S5). The gliding velocities were determined at different surface densities of each construct (Fig. 3). Upon decreasing the density of Myo IX head and Myo IX headΔext no changes in F-actin gliding velocity were observed. This is the characteristic behaviour expected for a processive motor molecule. In contrast, when the density of Myo IX headΔins was reduced, the velocity of filament gliding decreased. This is a behavior typical for nonprocessive motor molecules. Therefore, these observations suggest that the loop2 insertion is responsible for the processive behaviour of Myo IX. Notably, the maximum velocity of gliding filaments driven by Myo IX headΔins was somewhat slower (14.5 nm s^−1^) when compared to the velocities determined for Myo IX head (39.4 nm s^−1^) and Myo IX headΔext (41.8 nm s^−1^). Because deletion of the loop 2 insertion led to a nonprocessive behaviour of Myo IX, it might have been expected that the filament gliding velocity driven by Myo IX headΔins exceeds that of the processive constructs Myo IX head and Myo IX headΔext at high motor density. A possible explanation for this could be that deletion of the insertion might decrease either the step size or the coupling between ATP turnover and force production leading to a reduced maximal velocity.

### Influence of ionic strength on the processive behaviour of myosin IX head

**Figure 4:**
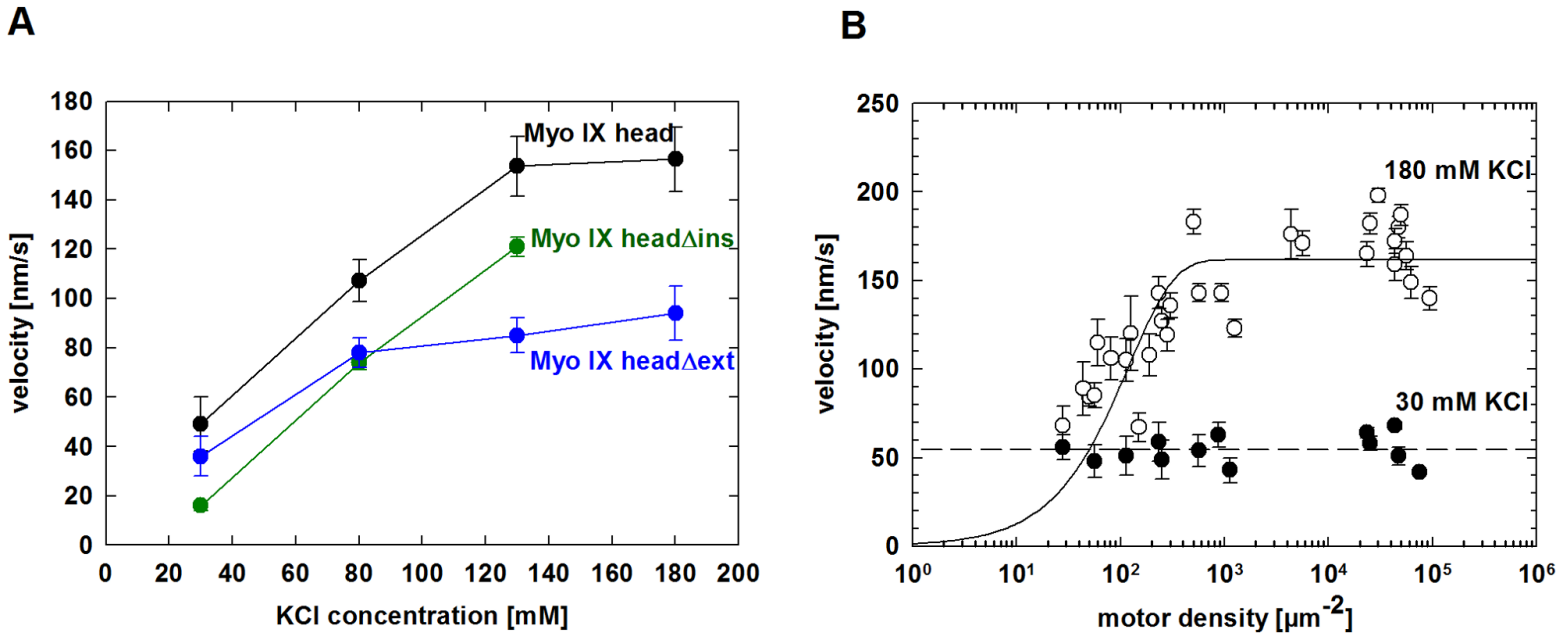
Influence of ionic strength on myosin IX head processivity. Filament gliding assays were performed as described in experimental procedures. **A:** Myo IX head (black), Myo IX headΔins (green) and Myo IX headΔext (blue) were immobilized specifically by biotin-streptavidin interaction on the surface of a flow cell. Velocity of F-actin gliding was determined at 30 mM, 80 mM, 130 mM and 180 mM KCl. **B:** Varying amounts of purified Myo IX head were immobilized on the surface of a flow cell. Velocity of F-actin gliding was determined at 30 mM KCl (closed circles) and 180 mM KCl (open circles), respectively. Errors presented are standard errors of varying numbers of tracked filaments.

Analysing the influence of ionic strength on Myo IX head motility might shed some light on the mechanism by which the loop2 insertion supports processive movement. Variation of ionic strength showed that at high motor density the filament gliding velocity increased with increasing concentration of KCl until the filaments finally dissociated (Fig. 4A). Myo IX head and Myo IX headΔext supported filament gliding up to 180 mM KCl and filaments dissociated at 200 mM KCl, whereas Myo IX headΔins supported gliding only up to 130 mM KCl. To test if higher gliding velocity could be a consequence of a loss of processivity, we analysed the gliding velocity powered by Myo IX head at low (30 mM KCl) and high (180 mM KCl) salt concentrations as a function of motor density on the surface (Fig. 4B). In contrast to the findings at low salt concentrations, gliding velocity varied with Myo IX head density under high salt conditions (Fig. 4B). This result demonstrates that high ionic strength abolishes the processive characteristics of Myo IX head motility and that electrostatic interactions of Myo IX head with the actin filament contribute to the processive behaviour of Myo IX.

### Quantification of the number of motor molecules required for movement of an actin filament

**Figure 5:**
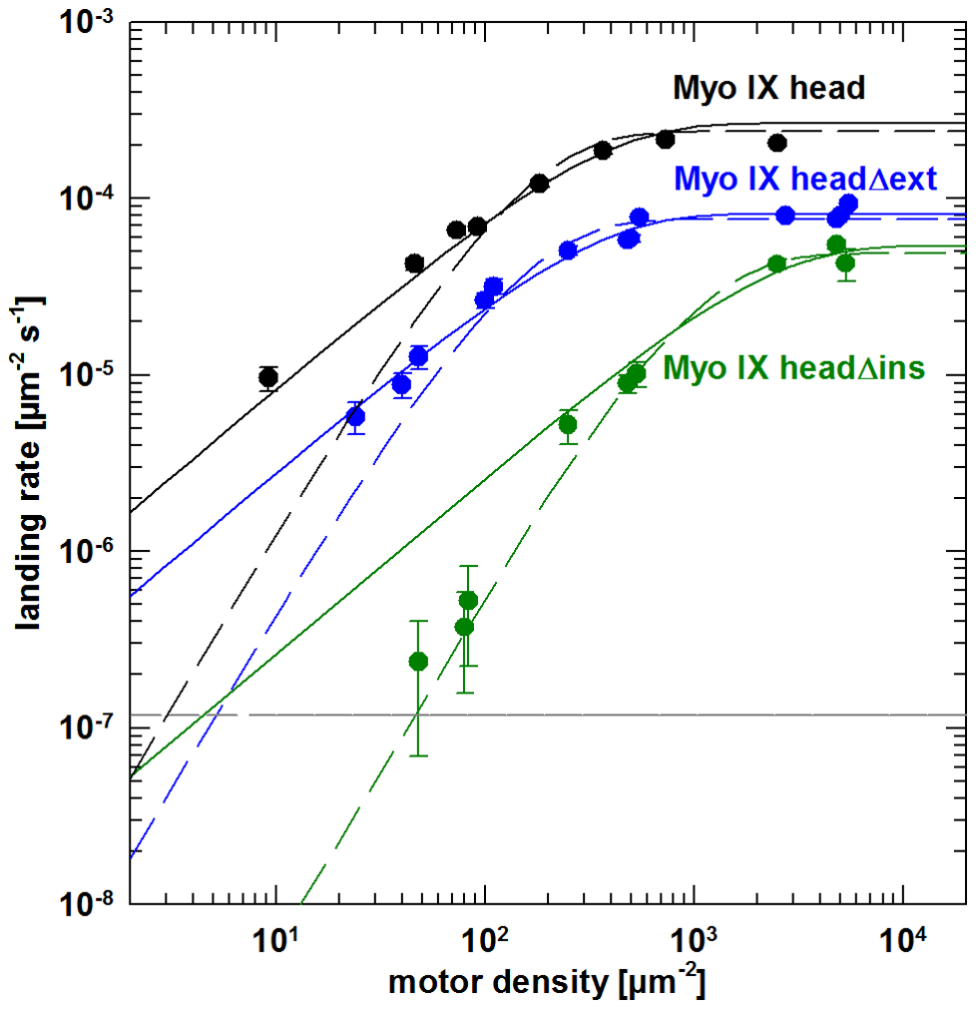
Actin filament landing rates as a function of myosin IX head construct density. Successful filament landing and gliding events were determined at varying motor densities on the surface for Myo IX head (black) Myo IX headΔins (green) and Myo IX headΔext (blue). Error bars represent the S.E.M. calculated from counting statistics (S.E.M.  =  landing rate/√n; n  =  number of landing events). For each data set, two theoretical curves were generated by non-linear least-squares fitting of parameters l_max_ and A of equation 3 with fixed n = 1 (solid lines) or n = 2 (dashed lines). Correlation coefficients indicate that n = 2 yields the best fit for headΔins, whereas head and headΔext are best fitted with n = 1 (head: R^2^_(n = 1)_ = 0.9845; R^2^_(n = 2)_ = 0.9370; headΔins: R^2^_(n = 1)_ = 0.9663; R^2^_(n = 2)_ = 0.9805; headΔext: R^2^_(n = 1)_ = 0.9729; R^2^_(n = 2)_ = 0.9268). The horizontal dotted grey line represents the experimental limit of detection given by the time and area of observation.

To determine the number of motor molecules required for movement of a single actin filament, landing assays were performed. In this assay F-actin was introduced along with ATP into flow cells with varying motor densities on their surfaces. The rates of filament landing and movement for at least three consecutive frames were estimated and plotted against the motor density (Fig. 5). The slope at low motor density represents the number *n* of motor molecules required for movement of a single filament. When more molecules are necessary to move actin, the landing rate drops more abruptly with decreasing motor density. Experimental data were fitted according to equation 3 with either *n* = 1 or *n* = 2. In the case of Myo IX head and Myo IX headΔext, there was good agreement with the theoretical curves for *n* = 1, which means that a single molecule is able to support filament gliding. On the other hand, a single molecule of Myo IX headΔins turned out not to be sufficient to generate movement. This result suggests that the construct lacking the loop2 insertion is no longer able to support processive movement.

### Direct observation of Qdot-labelled myosin IX head constructs moving along actin filaments

**Figure 6:**
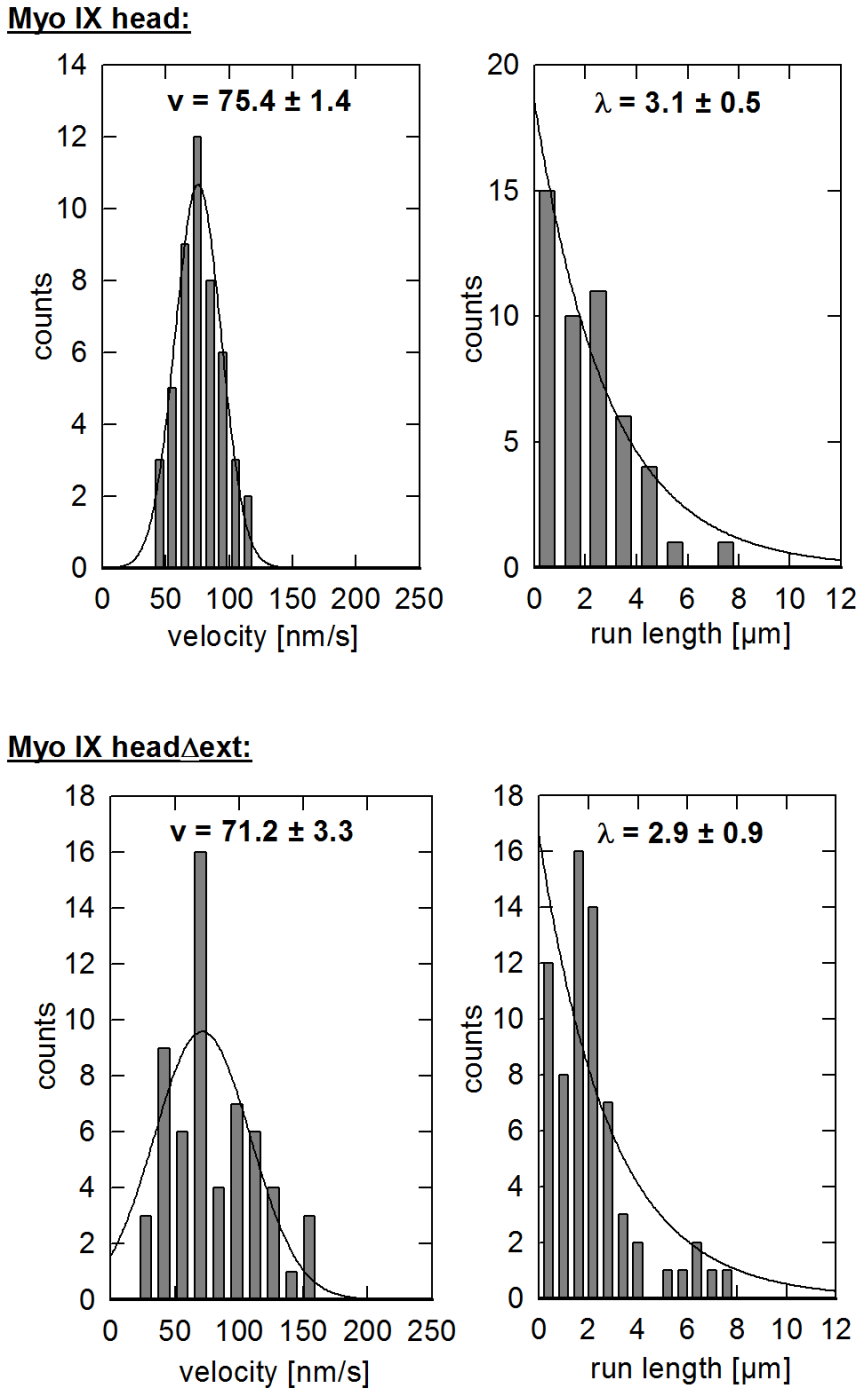
Movement of Qdot-labelled myosin IX head^(a)^ and myosin IX headΔext. Purified Myo IX head and Myo IX headΔext as indicated were labelled with streptavidin coated Qdots in a mixing ratio of 2 Qdots/Myo IX and introduced into a flow cell with immobilized actin filaments. Histograms of the velocity of moving Qdots fit a Gaussian distribution. Average run length of moving Qdots was determined by fitting the distribution to a function of an exponential decay as described in experimental procedures. The mean velocities (v) and run lengths (λ) are indicated. Exemplary movies (S6 and S7) can be found in supporting information. ^(a)^ published by Liao *et al.* [12], shown for comparative reasons.

In both the filament gliding and landing assays, the motor activities of Myo IX head constructs were analysed indirectly by monitoring the gliding of actin filaments. To get direct insight into Myo IX movement, stepping assays were performed utilizing Myo IX head constructs that were labelled with streptavidin coated Qdots. Both Myo IX head and Myo IX headΔext coupled Qdots were observed to move along actin filaments (movies S6 and S7). The observed velocities and run lengths are shown in Fig. 6 and Table 1. In both cases the mean velocity of 75 nm s^−1^ for Myo IX head and 71 nm s^−1^ for Myo IX headΔext, respectively, was higher than the filament gliding velocity driven by these constructs (39 nm s^−1^ and 42 nm s^−1^ respectively). Both constructs were able to move along actin filaments for considerable distances before dissociation with an average run length of 3.1 µm (Myo IX head) and 2.9 µm (Myo IX headΔext).

**Table 1:** Parameters of *in vitro* motility of different myosin IX head constructs.

	gliding assay	stepping assay	
construct	v ± S.E.M. [nm s−1]	v ± S.E.M. [nm s^−1^]	λ ±S.E.M.e [µm]
Myo IX head	39.4±1.5	75.4±1.4	3.1±0.5
Myo IX headΔins	14.5±0.3	n.d.	n.d.
Myo IX headΔext	41.8±3.1	71.2±3.3	2.9±0.9

F-actin gliding velocity v driven by different Myo IX head constructs is given (gliding assay). Mean velocity v and run length λ of Qdot-labelled Myo IX head and Myo IX headΔext moving along immobilized actin filaments (stepping assay) were determined from histograms shown in figure 6. (n.d.  =  not detectable).

Several binding events but no movement of Qdot labelled Myo IX headΔins could be observed even when Qdots were mixed with an excess of Myo IX headΔins. This finding again supports the notion that the loop 2 insertion is required for processive movement of the Myo IX head. Furthermore, the fact that no moving Qdots were detected with Myo IX headΔins even under conditions favouring the association of multiple myosin molecules indicates that the geometry of the multiple molecules on the surface of streptavidin coated Qdots does not allow for continuous movement along the actin filament. This further demonstrates that the loop2 of Myo IX is a critical determinant for processivity.

## Discussion

Class IX myosins are single-headed myosins reported to move processively along actin filaments [8]–[12]. Single headed myosin V was reported by Watanabe *et al.* of being also able to take multiple successive steps [24]. Therefore, these authors concluded that dimerisation of myosins is not essential for processivity, although it improves efficiency by increasing the number of successive steps. The mechanism by which myosin IX that differs significantly from myosin V in its kinetic parameters achieves single-headed processivity is not established, but it must differ from the hand-over-hand mechanism of double-headed myosins. In the present study we explored the roles of the N-terminal extension and the loop2 insertion, unique sequences of class IX myosins, in the processive behaviour of Myo IX.

Deletion of the N-terminal extension did not alter the processive behaviour of Myo IX movement. Its function may not primarily relate to motor properties. Previous work has suggested that this N-terminal extension has a folding topology similar to Ras-binding domains. However, it did not show binding to the small G-protein Ras [25]. Its function remains to be elucidated.

Replacement of the extended insertion at loop 2 with three glycine residues as a linker resulted in the loss of processive behaviour of Myo IX as determined by filament gliding and filament landing assays. Furthermore, in the stepping assay we were not able to observe directed movement of Qdots associated with Myo IX headΔins although we tried extensively. On the other hand, Qdots associated with Myo IX head or Myo IX headΔext showed directed movement along the F-actin track. Deletion of the loop2 insertion included the recently discovered calmodulin binding site at the N-terminus of the insertion, but not two consecutive lysine residues at the C-terminal end. Positively charged residues at the C-terminal end of loop 2 are found in several myosin classes and seem to contribute to actin affinity [22], [26], [27].

How might the loop2 insertion affect the motor properties of MyoIX? It had been proposed by several groups that the loop2 insertion of myosin IX might act as an actin tether to prevent dissociation from actin filaments [11], [17], [18], [20], [21], [22]. Our present results support this hypothesis. The tethering of Myo IX head to F-actin by the loop2 insertion can be overcome when the ionic strength is increased. This suggests that the interaction of the loop 2 insertion with actin filaments is at least in part electrostatic. The loop 2 insertion of class IX myosins is highly basic and the pI value of the loop2 insertion of *C. elegans* Myo IX is 10.8. The positively charged residues are likely to interact with the negatively charged surface of the actin filament. In accordance with this notion, we have shown previously that deletion of the loop2 insertion in rat Myo IXb causes a reduction in the affinity for F-actin and that the isolated rat Myo IXb loop2 insertion has a strong actin filament binding and bundling activity [22]. A similar function has also been assigned to the much shorter loop 2 of Myo5a. In the processive double-headed Myo5a the net charge of loop 2 has been shown to affect both actin affinity and processive run length [27]. Reducing the net positive charge of loop 2 decreased run length, whereas introducing additional positive charges increased the run length. Similarly, the positively charged K-loop of the microtubule-dependent kinesin KIF1A and the negatively charged C-terminus of tubulin were shown to enable single-headed processive movement of KIF1A [28], [29]. These examples suggest that tethering of a molecular motor to the track by electrostatic interaction might be a common mechanism to allow for single-headed processivity.

To allow for coordinated movement of single-headed Myo IX, the loop2 insertion could change conformation during the chemo-mechanical cycle. Major rearrangements in loop2 of Myo5a during the chemo-mechanical cycle have been observed by electron cryo-microscopy [30]. Alternatively, the loop2 insertion could force 1D-diffusion of Myo IX along the F-actin track. Directionality of movement could be established by the asymmetric interaction potential of myosin and actin [21]. A very similar mechanism has been postulated for the single-headed processive kinesin KIF1A [28]. Whatever the mechanism, the loop2 in class IX myosins evolved by increasing its length and adapting its function to enable characteristics of myosin processivity. Whether Myo IX acts as a processive motor *in vivo* remains to be seen.

## Materials and Methods

### Chemicals and reagents

Unless otherwise indicated all chemicals and reagents were purchased from Sigma-Aldrich (Steinheim, Germany).

### Construction of plasmids and generation of recombinant baculoviruses

Generation of recombinant baculovirus encoding the head of Myo IX has been described previously [12]. The Myo IX headΔins construct lacks the large basic insertion from amino acid residues 713–820. The deleted amino acid residues were replaced by a linker consisting of three glycine residues. The Myo IX headΔext construct encompasses amino acid residues 151–959. All constructs include a C-terminal avi-tag for specific biotination followed by a FLAG-tag for purification.

Plasmid pET21a-BirA for expression of biotin ligase in E. coli was a kind gift from Alice Y. Ting (MIT, Cambridge, USA). Myo IX and BirA DNA constructs were subcloned into the transfer vector pFastBac™1 (Invitrogen). Recombinant baculovirus DNA was generated by the Bac-to-Bac method as described previously and transfected into *Spodoptera frugiperda* (Sf9) cells [22]. Individual viruses were isolated by end point dilution and then amplified three times. Final virus titers were determined prior to infection of Sf9 cells for protein expression. The generation of recombinant baculovirus encoding rat calmodulin has been described previously [18].

### Protein expression, purification and modification

For preparation of Myo IX head, Myo IX headΔins or Myo IX headΔext constructs 400 ml of Sf9 cells (1×10^6^ cells ml^−1^) cultured in Grace's medium (Invitrogen) with 10% (v/v) fetal calf serum (PAN Biotech) and 0.2 mg ml^−1^ biotin were triple-infected with the corresponding Myo IX construct, rat calmodulin and biotin ligase recombinant baculoviruses. The multiplicity of infection was 4 for Myo IX head viruses, 8 for calmodulin virus and 2 for biotin ligase virus. Infected Sf9 cells were collected after 72 h and washed once with phosphate-buffered saline. The consecutive steps were performed at 4°C. Cells were resuspended in 30 ml lysis buffer (20 mM Tris-HCl, pH 7.4, 200 mM NaCl, 2 mM MgCl_2_, 1 mM EGTA, 10% (v/v) glycerol, 2 mM ATP, 0.1 mg ml^−1^ Pefabloc, 0.01 mg ml^−1^ leupeptin, 0.02 unit ml^−1^ aprotinin) and lysed by sonication (three times 30 pulses at 100% and a cycle of 0.6 with UP100H sonicator, Hielscher ultrasound technology). The homogenate was clarified by centrifugation at 170,000× *g* for 45 min. 4 µg ml^−1^ calmodulin was added to the cleared lysate before it was loaded onto a column of 0.4 ml of pre-equilibrated α-FLAG-M2-affinity-agarose. After passing the cleared lysate over the column three times for 90 min in total, the column was washed with 10 ml of lysis buffer and pre-equilibrated with 5 ml assay buffer (20 mM Hepes, pH 7.4, 30 mM KCl, 2 mM MgCl_2_, 1 mM EGTA, 10% (v/v) glycerol, 1 mM β-mercaptoethanol). Myosin was eluted with 0.05 mg ml^−1^ FLAG peptide in assay buffer. Protein concentration was determined by Bradford assay using BSA as a standard and purity was determined by gel densitometry. Purified proteins were stored at 4°C and analyzed within 2 days. Prior to each experiment proteins were centrifuged at 150,000× *g* for 10 min.

Actin was isolated from rabbit skeletal muscle as described by Pardee and Spudich [31]. Rat calmodulin was expressed in *E.coli* BL21 cells and purified by hydrophobic phenyl-sepharose chromatography [18]. Myosin II HMM was prepared according to Margossian and Lowey [32] and inactivated by treatment with N-ethyl-maleimide to produce NEM-HMM [33] for specific attachment of F-actin.

### Size distribution of myosin IX head

Size distribution analysis was performed by size exclusion chromatography. A volume of 200 µl of purified Myo IX head was loaded onto a pre-equilibrated Superdex 200 10/300 column (GE). AB buffer containing 130 mM KCl was used as running buffer at a flow-rate of 0.25 ml/min and absorption was monitored at 280 nm. In order to calibrate the column the elution volume of the following proteins was determined: ferritin (440 kDa), catalase (232 kDa), aldolase (158 kDa), albumin (66 kDa), carbonic anhydrase (29 kDa) and cytochrome C (12.4 kDa). To estimate the molecular weight the partition coefficient K_av_ was calculated from the void volume V_0_, the total bed volume V_t_ and the elution volume V_e_ of a certain protein in the following manner:
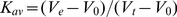
(Eq. 1)


### In vitro motility assay

Gliding filament assays were performed at room temperature, basically as described by Reck-Peterson *et al.* [34] with some modifications. Flow cells were prepared from a glass slide and a coverslip, separated by two strips of double-sided tape. The glass slide was precoated with 0.5 mg ml^−1^ BSA (Carl Roth GmbH, Karlsruhe, Germany) and the coverslip with 1% (v/v) nitrocellulose in iso-amylacetate. All reagents were prepared in assay buffer (AB) containing 25 mM imidazole, pH 7.4, 2 mM MgCl_2_, 10 mM dithiothreitol, 1 mM EGTA and 25 mM KCl. Reagents were introduced into the flow-cells and incubated for 2 min followed by washing with AB in the following order: 0.5 mg ml^−1^ biotinated BSA, 1% (w/v) pluronic F127, 0.5 mg ml^−1^ streptavidin, indicated amounts of myosin constructs, 6.5 nM phalloidin-tetramethyl rhodamine isothiocyanate (TRITC) labeled F-actin and finally 2 mM ATP including 0.5% (w/v) methylcellulose. The AB buffer used for all washing steps after myosin application contained additionally 10 µM calmodulin and an oxygen scavenging system (2.5 mg ml^−1^ glucose, 100 µg ml^−1^ glucose oxidase, 20 µg ml^−1^ catalase). The filament landing assay was performed in the same manner except that labeled F-actin was introduced in the presence of 2 mM ATP. Samples were observed with a Nikon TE-2000 microscope equipped with a 100x TIRF-objective and a Cascade II 512 camera (Photometrics). Movies were recorded with Nikon NIS-Elements software with a time interval of 10 s in experiments with Myo IX head and Myo IX headΔext or 20 s in experiments with Myo IX headΔins. In experiments analysing the influence of ionic strength the time interval was lowered to 5 s at 130 and 180 mM KCl. Filaments moving continuously for at least three frames were used to calculate gliding velocities by ImageJ software. Filament landing rates were determined by counting the filaments that were landing and moving for at least three frames. The velocity as a function of motor density was fitted by the following equation with V being the average filament velocity, f the duty ratio, d the motor density and A the mean interaction area surrounding a filament:
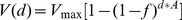
(Eq. 2)


### Data analysis of filament landing assays

To estimate the number of motor molecules required for binding and moving a single filament, a model originally described by Hancock and Howard was used to fit landing rate data [35]. Here the landing rate (l) is expressed as a function of maximum landing rate (l_max_), motor density (d), the mean interaction area (A) and the number of motor molecules (n) required to give a successful filament landing and gliding event (Eq. 3).
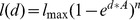
(Eq. 3)


Fits were generated with SigmaPlot 12 by non-linear regression with n = 1 or n = 2, with l_max_ and A as fitted parameters. Surface myosin densities given assume that every molecule introduced into the flow cell was adsorbed to the surface in a favourable orientation and that none got denatured. This calculation results in an uncertainty of motor density due to several reasons: (i) the number of active, *i.e.* non-denatured motor molecules is not known; (ii) a gradient of myosin density can be produced on the surface due to gradual binding of myosin while the sample is introduced into the flow cell; (iii) unspecific binding of motor molecules to the glass slide is possible. However, the number of motor molecules required for movement of one actin filament estimated from the filament landing assay is only marginally influenced by this existing systematic error in the calculation of motor density.

### Quantum dot stepping assay

Determination of stepping velocities of Quantum dots (Qdots) driven by different Myo IX head constructs was performed basically as described previously [12]. Flow cells were prepared as described for the *in vitro* motility assay. First, 1 mg ml^−1^ NEM-HMM was introduced and incubated for 2 min. Then, the surface was blocked by the addition of 1% (w/v) pluronic F127. TRITC-phalloidin-labeled F-actin (6.5 nM) was introduced, and flow cells were washed immediately with AB buffer. Biotinated Myo IX head was preincubated with 10 nM streptavidin-coated Qdots 525 (Invitrogen) in a molar ratio of 2 Qdots/Myo IX head for 3 min in the presence of 2 mM ATP. At this mixing ratio 91% of Qdots should not carry more than one Myo IX head as calculated assuming a binomial distribution. After introduction of this premix into the flow cell, images were recorded every 5 s. The characteristic run length of Qdots moving along actin filaments was calculated by fitting the data to equation 4 using SigmaPlot 10.0 software with *P*(x) being the probability of a Qdot moving a distance × along the F-actin track.
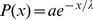
(Eq. 4)


## Supporting Information

Movie S1 **Filament gliding assay using multimeric Myo IX head fractionated by size exclusion chromatography.** Fractions containing multimeric Myo IX head (MW ≥ 1300 kDa) with a concentration of 20 nM were collected from SEC and used for *in vitro* motility assay in the gliding-setup.
(MPG)

Movie S2 **Filament gliding assay using monomeric MyoIX head fractionated by size exclusion chromatography.** Fractions containing monomeric MyoIX head (MW = 181±4.5 kDa) with a concentration of 20 nM were collected from SEC and used for *in vitro* motility assay in the gliding-setup.
(MPG)

Movie S3 **Filament gliding by Myo IX head.** Purified Myo IX head (200 nM) was immobilized on the surface. F-actin was introduced under rigor conditions and the assay was started by the addition of 2 mM ATP.
(MPG)

Movie S4 **Filament gliding by Myo IX headΔext.** Purified Myo IX headΔext (200 nM) was immobilized on the surface. F-actin was introduced under rigor conditions and the assay was started by the addition of 2 mM ATP.
(MPG)

Movie S5 **Filament gliding by Myo IX headΔins.** Purified Myo IX headΔins (200 nM) was immobilized on the surface. F-actin was introduced under rigor conditions and the assay was started by the addition of 2 mM ATP.
(MPG)

Movie S6 **Movement of Qdot-labeled Myo IX head along F-actin.** F-actin was immobilized on the surface. Qdot-labeled Myo IX head along with 2 mM ATP was introduced into the flow-cell.
(MPG)

Movie S7 **Movement of Qdot-labeled Myo IX headΔext along F-actin.** F-actin was immobilized on the surface. Qdot-labeled Myo IX headΔext along with 2 mM ATP was introduced into the flow-cell.
(MPG)
